# Communication skills of medical students: survey of self- and external perception in a longitudinally based trend study

**DOI:** 10.1186/s12909-020-02049-w

**Published:** 2020-05-11

**Authors:** Joachim Graf, Teresa Loda, Stephan Zipfel, Annette Wosnik, Daniela Mohr, Anne Herrmann-Werner

**Affiliations:** 1 University Hospital Tuebingen, Institute for Health Sciences, Section of Midwifery Science, Hoppe-Seyler-Strasse 9, 72076 Tuebingen, Germany; 2 Internal Medicine, Department of Psychosomatic Medicine and Psychotherapy, University Hospital Tuebingen, Osianderstrasse 5, 72076 Tuebingen, Germany; 3 Medical Faculty Tuebingen, Dean’s Office for Students’ Affairs, Geissweg 5, 72076 Tuebingen, Germany; 4 Medical Faculty Tuebingen, Interdisciplinary Training Centre DocLab, Elfriede-Aulhorn-Strasse 10, 72076 Tuebingen, Germany

**Keywords:** Communication skills, OSCE, Trend study, Self- and external perception, Empathy

## Abstract

**Background:**

As good communication skills are crucial for doctor-patient interactions, it is recommended to incorporate them in medical school programs from the very beginning. On this basis medical schools in Germany introduced the OSCE (objective structured clinical examination) to examine and by this foster learning of communication skills as assessment drives learning. The aim of the study was to examine the development of the communication skills of medical students during an OSCE to investigate how communication competence has developed between different student cohorts.

**Methods:**

This study is a longitudinal trend study based on seven semester-cohorts, examining the communication skills of medical students in the OSCE both from the perspective of students and from the viewpoint of standardized patients (SP). Altogether, 1027 students from seven semester cohorts were asked to rate their own communication skills (self-perception) before the OSCE exam started. Here, sub-analyses were performed to outline a potential influence of previous history-taking group participation. The SP evaluated the students’ communication skills in external perception during the OSCE exam at each station with history-taking or physical examinations. The communication skills in both groups were ascertained in the dimensions of empathy, content structure, verbal expression, and non-verbal expression.

**Results:**

Only in the dimension of non-verbal expression could a statistically significant change be found in students’ self-perception over the years. Notably, the rating of communication skills as self-rated by the students has risen constantly, whereas they deteriorated from the perspective of standardized patients (SP). It has also been found that previous history-taking courses have a positive influence on the structural dimension of communication skills in particular.

**Conclusions:**

The results of this study support conclusions of other studies which also suggest differences between self- and external perception of medical students’ communication skills. Nevertheless, students showed good overall communication skills in the four dimensions of empathy, content structure, verbal expression, and non-verbal expression, as demonstrated in a longitudinal trend study over seven semesters. However, we noted that externally rated empathy levels declined over the semester cohorts, suggesting the need for new priorities to be set in student teaching.

## Background

### Communication skills of physicians

In order to be a ‘good doctor’, physicians require not only clinical and scientific knowledge, but also excellent communication skills to ensure a good doctor-patient relationship, which is associated with better patient safety and treatment efficiency [1–6]. Patients should be involved as partners in the diagnostic process and subsequent treatment to encourage them to take responsibility for their own health and to improve compliance and engagement in an efficient patient-based health service [7–9]. It is the physician’s responsibility to foster this process of ‘shared decision making’ by utilizing good communication skills [10, 11]. Following the Kalamazoo I Consensus Statement, there are seven essential sets of communication tasks which are relevant to physician-patient communication: (1) build the doctor-patient relationship; (2) open the discussion; (3) gather information; (4) understand the patient’s perspective; (5) share information; (6) reach agreement on problems and plans; and (7) provide closure [12]. The competences necessary for the application of the Kalmazoo criteria can be taught in medical curricula and can be examined in OSCE exams (objective structured clinical examination) [13].

### Communication skills of medical students

As good communication skills are crucial for doctor-patient interactions, it is recommended to incorporate them in medical school programs from the very beginning [14, 15]. Despite ongoing debate about the importance of improving medical students’ and fully trained physicians’ communication skills, structured communication skills (e.g., for the training of history-talking and teaching of physical examination [16]) are still under-provided in Germany [17–22]. Since introduction of the latest medical licensure act in Germany (2002), which requires training and examination of social, communications, and interpersonal skills in the teaching of prospective physicians, German medical faculties are faced with the challenges of implementing practical examinations and reducing the influence of written and oral exams [23, 24]. As in other universities worldwide, medical schools in Germany introduced the OSCE to examine and thus foster learning of communication skills as assessment drives learning [25]. The OSCE was first described by Harden et al. in 1975 and has been used in the United States as an examination procedure since the 1980s [26–28]. In Germany, OSCE examinations have been held since the late 1990s. At our own faculty in Tübingen, the OSCE has been an integral part of the curriculum since 2004 [29].

The OSCE exam is a circuit of brief examinations in which the students must demonstrate their communication skills and practical abilities [30, 31]. In Tuebingen, the OSCE is held at the end of the third year, and students complete different station-types, including history-taking or physical examinations (both with standardised patients (SP)). At each station, an examiner evaluates the performance of the students [29, 32, 33]. In order to prepare medical students for this OSCE and to improve general doctor-patient communication, the Medical Faculty of Tuebingen offers a longitudinal communication curriculum (*iTüpFerl*) that accompanies traditional teaching. It starts in the first year with basic skills such as history-taking and feedback provision and progresses with more advanced classes dealing with such issues as delivering bad news or making inter-professional ward rounds [32, 34]. In addition, students can voluntarily take part in history-taking groups where usually around six to eight students meet weekly with one or two student tutors to take medical histories of inpatients and receive structured feedback on communication skills as well as student-patient interaction [18, 22]. Against the background of changing requirements regarding the curricular significance of communicative competences, changing teaching formats, and the socio-demographic structure of students with regard to age and gender, the evaluation of communication teaching’s efficacy in different student cohorts is of relevance. Although there have been various attempts to make communication skills of medical students in the OSCE exam measurable, we have been unable to find any longitudinal trend studies looking at this vital issue [32, 35, 36], as well as there is only a lack of longitudinal studies for measurement of students communicative skills’ development [37] . Furthermore, there is a variety of methodological and psychometric quality of assessing communication competences in present in literature [38]. On this basis, the present study wants to contribute a different perspective on the discussion by using longitudinal measurement. Literature suggests that communication skills of medical students are evaluated better in external assessment than in self-assessment but it remains open to what extend this influences competencies on the various dimensions of communicational skill [39, 40].

### Aims

The aim of the study was to examine the development of the communication skills of medical students during an OSCE as part of a longitudinal trend study in order to investigate how the communication competence has developed between different student cohorts. The development of the communicative skills were presented both from the perspective of students and from the viewpoint of SP. Furthermore, it will be analyzed whether the self-reported communication competency is dependent on past history-taking courses.

## Methods

### Study design

This study is a longitudinal trend study based on seven semester-cohorts, examining the communication skills of medical students from Tuebingen University at the end of the sixth semester. Trend studies (also called replicative surveys) represent the third subtype of longitudinal analyses (in addition to cohort and panel studies). A trend study samples different groups of people at different points in time but in the same situation and from the same population. The aim is to demonstrate the development of skills or attitudes in social groups like medical students, whereby not the individual but the whole group is focalized. While in cohort studies the same persons are interviewed at regular intervals (e.g., the same medical students in the course of studies, in the first, second, and other semesters), trend studies pursue the target to survey different persons of the same population at regular intervals (e.g., the students of the sixth semester in an OSCE looking at several consecutive OSCEs every half-year). So, trend studies use cross-sections at two or more points in time to examine change over time within a population [41–43]. The study design is also described in another paper of our working group [32]. According to the specifications of the University and University Hospital in Tuebingen, approval by the responsible ethics committee was not necessary because no patients were interviewed. The theoretical framework of OSCE in Tuebingen is based on recommendation in literature with a maximum score of 25 points to be awarded at each station [44]. New stations are created by the study coordinators of each subject and then checked by the overall coordinators as part of a review process. In order to meet the high quality standards and ensure a consistently high level of validity throughout the semesters, all newly developed stations undergo a communicative validation process involving all participants. In order to increase the communicative competences, the Kalamazoo criteria were taken into account in the preparation of all OSCE stations so that at least one of the defined criteria is examined in each station in addition to the subject-related competences.

### Survey details

A few days before the two-day OSCE examination, a training day is held, during which the SPs discuss their roles and the evaluation concept is explained to them. In order to ensure consistent quality, a simulation patient programme is linked to the Tübingen Medical Faculty. The individual patients are used in various communication courses during the semester to improve student communication skills. As a rule, we use the same SPs during all OSCE exams.

Students and SP were asked to rate students’ communication skills during the OSCE. All students who completed the OSCE between 2011 and 2014, as well as all SP who were deployed as actors in the OSCE during the same period, were included in the study. The students rated their own communication skills (self-perception) before the OSCE exam started. The SP evaluated the students’ communication skills in external perception during the OSCE exam at each station with history-taking or physical examinations. Both students and SP completed standardized uniform questionnaires to rate the communication dimensions empathy, structure, verbal expression, and non-verbal expression on a five (SP) and a six-point Likert scale (students). On the self-perception scale, 1 reflected ‘completely disagree’ and 6 ‘completely agree’. In the external rating of skills, 1 reflected the worst performance and 5 the best (see Tables 1 and 2). The definition of the various dimensions results from the items in the questionnaire, as shown in Table 1: For example, an empathic communication is reflected by students answering appropiately to the verbal and non-verbal cues and needs of their counterpart. The questionnaires were developed by our working group. In the process of development, we asked 20 students to fill out the questionnaires (10 students before and 10 students after completing the OSCE). Afterwards, the questionnaires were discussed and reflected within an interprofessional team (physicians, health scientists and medical students). After that the questionnaires were completed by 10 students and 10 physicians who had previously been asked to complete a mini OSCE consisting of 4 content stations in order to correlate the self-assessment with the individual OSCE grades in the context of questionnaire’s validation.

**Table 1:** Items of self-perception (students)

Item: Right now, I feel able to …	Rating
a) … answer sympathetically to the verbal and non-verbal cues and needs of my counterpart (empathy).	1 = completely disagree 2 = rather disagree 3 = partly accept 4 = rather agree 5 = agree 6 = completely agree
b) … organize a conversation coherently and direct the flow of the conversation (structure).	
c) … adapt my manner to my counterpart in wording, voice modulation, speech rate, etc. (verbal expression).	
d) … motivate my counterpart in the conversation by using non-verbal techniques (non-verbal expression).

**Table 2:** Items of external perception (standardized patients)

Item	1 2 3 4 5	Item
a) The student does not respond to the obvious (verbal and nonverbal) cues and needs from me as a SP and/or responds inappropriately (empathy).		a) The student always responds to obvious (verbal and nonverbal) cues and needs from me as a SP and/or responds appropriately (empathy).
b) The conversation is not organized recognizably; the student acts incoherently or I as SP have to set the course of the conversation (structure).		b) The conversation is excellently organized. The student’s approach shows that the (s)he is able to direct the conversation (structure).
c) The student communicates inappropriately with me as a SP (e.g., choice of words, volume) and/or communicates in a way that makes it impossible to understand him/her (verbal expression).		c) The student communicates appropriately with me as a SP (e.g., choice of words, volume) and/or communicates in a way that makes it easy for me to understand him/her (verbal expression).
d) The student does not manage to involve me as SP with his/her non-verbal expression and frustrates me and/or antagonizes me (non-verbal expression).		d) The student successfully involves me as a SP in the communication with his/her non-verbal expression and/or motivates me to participate (non-verbal expression).

The students were informed before the OSCE about the objectives of the study. They were asked to evaluate their own communication competences and were also informed that their competences would also be assessed by the SP. The students were enlightened that the communication assessments are not included in the examiners’ grading and are collected separately from the performance at the respective examination stations only for research reasons. Precisely because the assessment had no influence on the grading, the students did not receive feedback on their respective performance by default. This was also not possible due to the ananomymised survey: the students were initially given a sheet with the same numerical codes for each SP, with the instruction to give each SP a code for the rating sheets before the start of the examination. This procedure enabled us to assign all the questionnaires of the external and the self-evaluation questionnaires after completion of the OSCE. However, it was no longer possible to assign them to a specific student name.

### Statistics

First, we carried out a frequency analysis in order to identify the descriptive characteristics of the data. Subsequently, we conducted paired t-tests for independent samples. We conducted ANOVA with the data from self- and external perception to identify any significant relationship between the first and the last cohort in the four dimensions of communication and to identify differences between the individual semester cohorts. Next, sub-analyses were performed in the dimension of self-perception to outline a potential influence of previous history-taking group participation. Some of the medical students participated in an optional undergraduate course in amnestic groups together with psychologist students, which is described elsewhere [45]. For this, an unpaired t-test was performed between students with previous history-taking group participation (intervention group) and those without such structured additional teaching experience. Last, a mean value analysis of the four combined communication dimensions was performed in each semester to compare the developments for self- and external perception (unpaired t-test). In all analyses, a *p*-value of < 0.05 was considered to be statistically significant (α = 0.05). For data processing, MS Excel 2010 and SPSS 21 were used.

## Results

### Student population: socio-demographic characteristics

We recruited 1027 students from seven semester cohorts (summer semester 2011 through summer semester 2014). The average age of students across all seven cohorts was 24.9 ± 3.85 years. The gender distribution of the total student population was 60% female and 40% male (for further details see Table 3).

**Table 3:** Characteristics of the student population: age and gender

Semester	Number of Students	Gender Distribution		Age: Mean (Range (Min; Max)) [SD]
		***Male***	***Female***	
2011	162	32% (*n* = 52)	68% (*n* = 110)	24.86 (27 (21;48)) [4.19]
2011–2012	168	42% (*n* = 71)	58% (*n* = 97)	25.40 (38 (21;59)) [4.24]
2012	148	34% (*n* = 51)	66% (n = 97)	24.38 (32 (20;52)) [3.61]
2012–2013	81	44% (*n* = 36)	56% (*n* = 45)	25.93 (25 (21;46)) [4.66]
2013	150	40% (*n* = 60)	60% (*n* = 90)	24.75 (13 (21;34)) [3.03]
2013–2014	165	47% (*n* = 77)	53% (*n* = 88)	25.29 (33 (19;52)) [3.98]
2014	153	41% (*n* = 63)	59% (n = 90)	23.7 (19 (20;39) [2.93]
**Total**	1027	40% (*n* = 410)	60% (*n* = 617)	24.84 (24) [3.85]

### Self-perception of communication skills of the students

Table 4 shows results of sub-group analyses of communication skills in self-perception. In general, students rated their communication skills in all dimensions as good. Students rated their skills in the dimension of verbal expression highest (average score 4.41), closely followed by the dimensions of structure (mean = 4.38) and empathy (mean = 4.37). The dimension of non-verbal expression was rated lowest (mean = 4.11). All four dimensions improved slightly over time, although there was no significant change between the first and the last semester cohort when using the paired t-test. Only in the dimension of non-verbal expression could a statistically significant change be found (ANOVA, *p* = 0.006). The subgroup analyses between students with and without previous history-taking group participation rendered a statistically significant difference in favor of the students with previous history-taking group participation in the dimension of structure. Here, the students with previous history-taking group participation rated their communication skills much better than the students in the control group (mean 4.51 vs. 4.33, *p* = 0.02).

**Table 4:** Sub-group analyses of communication skills in students’ self-perception

Semester		Empathy	Structure	Verbal Expression	Non-verbal Expression
2011: Mean (Median) [SD]		4.36 (5) [1.15]	4.34 (5) [1.11]	4.48 (5) [1.10]	4.07 (4) [1.18]
2011–2012: Mean (Median) [SD]		4.21 (4) [1.08]	4.25 (4) [1.14]	4.25 (4) [1.16]	3.92 (4) [1.02]
2012: Mean (Median) [SD]		4.43 (5) [1.21]	4.45 (5) [1.10]	4.51 (5) [1.32]	3.97 (4) [1.09]
2012–2013: Mean (Median) [SD]		4.04 (4) [1.18]	4.12 (4) [1.39]	4.15 (5) [1.19]	3.95 (4) [1.26]
2013: Mean (Median) [SD]		4.36 (4) [1.01]	4.37 (4) [1.00]	4.36 (4) [1.04]	4.22 (4) [1.08]
2013–2014: Mean (Median) [SD]		4.59 (5) [1.16]	4.52 (5) [1.17]	4.61 (5) [1.28]	4.25 (4) [1.12]
2014: Mean (Median) [SD]		4.45 (5) [1.12]	4.50 (5) [1.11]	4.43 (5) [1.10]	4.32 (4) [1.17]
2011–2014/ overall: Mean (Median) [SD]		4.37 (5) [1.15]	4.38 (5) [1.14]	4.41 (5) [1.19]	4.11 (4) [1.14]
	Difference between 2011 and 2014	−0.08 [95%-CI: −0.34; 0.16]	− 0.17 [95%-CI: − 0.41; 0.08]	0.047 [95%-CI: − 0.2; 0.29]	−0.25 [95%-CI: − 0.51; 0.01]
	*p*-value (differences between 2011 and 2014)	0.4972	0.1873	0.7039	0.0618
	Levene’s test	*p* = 0.257	*p* = < 0.0001	*p* = 0.40	*p* = 0.40
	ANOVA (all semester cohorts)	F (6; 1020) = 2793	F (6; 2035) =3900	F (6; 1022) = 2184	F (6; 1020) = 3038^a^
	ANOVA, *p*-value	0.11	0.001	0.42	0.006^a^
	Students with anamnesis group (*n* = 258): Mean (Median) [SD]	4.46 (1.12) [5]	4.51 (1.10) [5]	4.42 (1.18) [5]	4.21 (1.17) [4]
	Control group (*n* = 726): Mean (Median) [SD]	4.34 (1.16) [5]	4.33 (1.08) [4.5]	4.40 (1.19) [5]	4.08 (1.12) [4]
	*p*-value (differences between anamnesis group and control group)	0.12	0.02^a^	0.81	0.13

^a^ statistically significant difference

### External perception by standardized patients

Table 5 shows results of sub-group analyses of communication skills in external perception. The trend of the external perception of communication skills as rated by the SP was different from the self-perception of the students. In total, *n* = 8484 communication sheets were analyzed: each student (*n* = 1027) was assessed on average by 8.24 SP. Through all semester cohorts, SP rated the dimension of verbal expression best (mean = 4.29), followed by empathy (mean = 4.27) and non-verbal expression (mean = 4.19), whereas the dimension of structure was rated lowest (mean = 4.12). The external perception of all four skills deteriorated over the semester cohorts. The dimension of empathy deteriorated most severely: we found a statistically significant worsening between the first (summer semester 2011) and the last cohort (summer semester 2014). There was no homogeneity of variances on Levene’s test, so we could not perform ANOVA.

**Table 5:** Sub-group analyses of communication skills in SP’s external perception

Semester	n	Empathy	Structure	Verbal Expression	Non-verbal Expression
2011: Mean (Median) [SD]	1468	4.21 (4) [0,74]	4.04 (4) [0.87]	4.18 (4) [0.82]	4.14 (4) [0.83]
2011–2012: Mean (Median) [SD]	1483	4.37 (4) [0.70]	4.23 (4) [0.8]	4.37 (5) [0.74]	4.24 (4) [0.79]
2012: Mean (Median) [SD]	1322	4.34 (4) [0.75]	4.12 (4) [0.85]	4.33 (5) [0.82]	4.22 (4) [0.86]
2012–2013: Mean (Median) [SD]	807	4.25 (4) [0.79]	4.01 (4) [0.85]	4.17 (4) [0.87]	4.09 (4) [0.87]
2013: Mean (Median) [SD]	1407	4.26 (4) [0.63]	4.21 (4) [0.71]	4.37 (4) [0.67]	4.24 (4) [0.69]
2013–2014:Mean (Median) [SD]	1623	4.21 (4) [0.74]	4.09 (4) [0.78]	4.28 (4) [0.76]	4.17 (4) [0.78]
2014:Mean (Median) [SD]	374	4.10 (4) [0.91]	4.06 (4) [0.92]	4.21 (4) [0.91]	4.08 (4) [0.93]
2011–2014/ overall: Mean (Median) [SD]	8484	4.27 (4) [0.73]	4.12 (4) [0.82]	4.29 (4) [0.79]	4.19 (4) [0.81]
Difference between 2011 and 2014		0.11 [95%-CI: 0.02; 0.2]^a^	−0.02 [95%-CI: − 0.12; 0.08]	−0.02 [95%-CI: − 0.12; 0.08]	0.06 [95%-CI: − 0.03; 0.16]
p-value		0.0147^a^	0.7440	0.6530	0.2062

^a^ statistically significant difference

### Coherence between self-perception and external perception

**Fig. 1:**
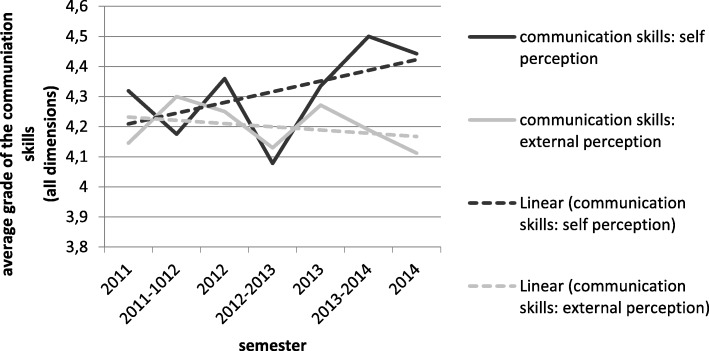
Change of communication skills in self- and external perception

We performed a mean value analysis of the combined four communication dimensions for self- and external perception in each semester. The results are shown in Fig. 1 (real course and trend). The self-perception of communication skills improved, while the external perception of these skills worsened. Overall, the difference between the two widened over time. In six of seven semesters, the differences between self- and external perception was statistically significant; in five semesters it was highly significant (see Table 6). We could not find any statistical correlation between the two spheres when matching them on an individual level.

**Table 6:** Statistical analysis between self- and external perception

Semester	Communication Skills: Self-Perception		n	Communication Skills: External Perception		n	Difference	95%-CI	*p*-value (α = 0,05)
	**Mean**	**SD**		**Mean**	**SD**				
**2011**	4.32	1.14	810	4.15	0.82	5854	0.173	0.109; 0.237	**< 0**.**0001**
**2011–2012**	4.17	1.11	818	4.3	0.76	5908	−0.125	− 0.185; − 0.066	**< 0**.**0001**
**2012**	4.36	1.18	740	4.25	0.82	5276	0.1096	0.042; 0.177	**< 0**.**0001**
**2012–2013**	4.08	1.36	405	4.13	0.85	3228	−0.051	−0.147; 0.044	0.2890
**2013**	4.33	1.03	759	4.27	0.68	5628	0.064	0.008; 0.118	**0**.**0237**
**2013–2014**	4.5	1.18	818	4.19	0.77	6492	0.311	0.251; 0.371	**< 0**.**0001**
**2014**	4.44	1.12	762	4.11	0.92	1496	0.331	0.244; 0.417	**< 0**.**0001**

## Discussion

### Principal findings

Overall, the communication skills of students in the dimensions of empathy, structure, verbal expression, and non-verbal expression can be described as acceptable when rated by students themselves and SP. However, there remains potential for improvement across these dimensions. Notably, the rating of communication skills has risen constantly as self-rated by the students, whereas they deteriorated from the perspective of SP. In this case, the external perception might be more reliable as several SP rated the same student, whereas the self-perception was only rated by the student him/herself. It has also been found that previous history-taking courses have a positive influence on the structural dimension of communication skills in particular.

### Relevance and limitations

This study examines communication skills in medical students. The strengths include the large number of students (*n* = 1047) and the comparison of self-perception and external perception. Additionally, we included four dimensions of communication skills (empathy, structure, verbal expression, and non-verbal expression), while other studies only analyzed the dimension of empathy [46, 47]. This is to our knowledge the first longitudinal trend study examining the communication skills of medical students. A possible confounder in this study is the point of time of the survey: we gathered our data in the busy and tense atmosphere of an examination. It is possible that both students and SP would have rated the communication skills differently in a normal classroom situation. We have decided to interview students before and not after the exam because we know from various preliminary studies that students often assess themselves significantly worse than they really are after an exam due to the exam situation they have experienced. All students were already familiar with the questionnaires from the pre-examination courses for preparing for the OSCE, so we expected more objective results if the students were interviewed before the examination. However, there may be a limitation here, since only the expected self-evaluation and not the experienced self-evaluation could be compared with the experienced external evaluation. Since the same evaluation forms were used throughout the entire study period and the students were informed in advance that the communication evaluation was explicitly not included in the examiners’ grade evaluation, we do not expect a confounder here. Possible power discourses might also have had a limiting effect: the response behavior of the students might have been influenced by the fact that they were asked to fill in the questionnaires by the examining authorities. It is also unclear whether the SPs were also influenced by subjective factors such as sympathy when completing the questionnaires. Another limitation is the different scaling of the questionnaires used: students rated their skills on a six-point Likert scale, while the SP used a questionnaire based on five points. Despite this, it was possible to determine the level of communication skills and to identify certain trends reliably.

### Comparison with prior work

The results of this study confirm conclusions of other studies that also suggest differences between self- and external perception of medical students’ communication skills [39, 40]. Our results agree with other authors who also find differences between self-reported and external reported skills by SP, whereby other papers only focalize single dimensions of communication skills such as empathy [46]. Our study also confirms other studies in relation to sustainability of communication skills acquisition: in this longitudinal analysis, previous history-taking group participation had a positive influence on communication skills when self-assessed, since students in the anamnesis group showed better performance in the dimension of structure. Other studies showed higher empathic tendencies after communication skills training [47]. Dong et al. showed longitudinal effects of medical students’ communication skills on future performance [48]. There are no other longitudinal trend studies focalizing development of students’ communication skills in different cohorts, which exacerbates integration of the present results in the research. This decline in empathy over time when rated by the SP is an interesting finding. It has previously been reported that empathy declines over the years of medical studies within the same individual [49], but here we found a collective reduction of empathy over different semesters (from 2011 to 2014) as rated by SP. This may be related to the decrease in age of medical students: in summer semester 2011 the average age of students was 24.86 years; by summer semester 2014 it had decreased to 23.7 years. Due to the conversion to an eight-year secondary school system and the abolition of mandatory military and civil service, German students are now much younger at enrolment in university than a few years ago. Another reason for the decrease in the external empathy rating may lie in the professionalization of the SP whose preparatory training has been intensified in recent semesters. Furthermore, many of our SP have worked within the OSCE exam for many years and may assess the students’ performance more critically due to their more extensive experience than they did a few years earlier.

## Conclusion

Strong communication skills are important for prospective physicians and should be taught as early as possible in medical training as a key component of the curriculum of medical schools. Medical students in Tuebingen showed good overall communication skills in the four dimensions of empathy, content structure, verbal expression, and non-verbal expression, as demonstrated in a longitudinal trend study over seven semesters. However, we noted that externally rated empathy levels declined over the semester cohorts, suggesting the need for new priorities in student teaching.

## Data Availability

The datasets used and/or analysed during this study are available from the corresponding author on reasonable request.

## Abbreviations

OSCE: Objective measurement of learning in clinical examinations
SP: Standardised patient
